# Microfluidic chip combined with magnetic-activated cell sorting technology for tumor antigen-independent sorting of circulating hepatocellular carcinoma cells

**DOI:** 10.7717/peerj.6681

**Published:** 2019-04-01

**Authors:** Xuebin Wang, Liying Sun, Haiming Zhang, Lin Wei, Wei Qu, Zhigui Zeng, Ying Liu, Zhijun Zhu

**Affiliations:** 1 Department of General Surgery, Beijing Friendship Hospital, Capital Medical University, Beijing, P. R. China; 2 Liver Transplantation Center, National Clinical Research Center for Digestive Diseases (NCRC-DD), Beijing Friendship Hospital, Capital Medical University, Beijing, P. R. China; 3 Beijing Key Laboratory of Tolerance Induction and Organ Protection in Transplantation, Beijing Friendship Hospital, Capital Medical University, Beijing, P. R. China

**Keywords:** Microfluidic chip, Antigen-independence, Hepatocellular carcinoma, Circulating tumor cell sorting, MACS technology

## Abstract

**Purpose:**

We aimed to generate a capture platform that integrates a deterministic lateral displacement (DLD) microfluidic structure with magnetic-activated cell sorting (MACS) technology for miniaturized, efficient, tumor antigen-independent circulating tumor cell (CTC) separation.

**Methods:**

The microfluidic structure was based on the theory of DLD and was designed to remove most red blood cells and platelets. Whole Blood CD45 MicroBeads and a MACS separator were then used to remove bead-labeled white blood cells. We established HepG2 human liver cancer cells overexpressing green fluorescent protein by lentiviral transfection to simulate CTCs in blood, and these cells were then used to determine the CTC isolation efficiency of the device. The performance and clinical value of our platform were evaluated by comparison with the Abnova CytoQuest™ CR system in the separating of blood samples from 12 hepatocellular carcinoma patients undergoing liver transplantation in a clinical follow-up experiment. The isolated cells were stained and analyzed by confocal laser scanning microscopy.

**Results:**

Using our integrated platform at the optimal flow rates for the specimen (60 µl/min) and buffer (100 µl/min per chip), we achieved an CTC yield of 85.1% ± 3.2%. In our follow-up of metastatic patients, CTCs that underwent epithelial–mesenchymal transition were found. These CTCs were missed by the CytoQuest™ CR bulk sorting approach, whereas our platform displayed increased sensitivity to EpCAM^low^ CTCs.

**Conclusions:**

Our platform, which integrates microfluidic and MACS technology, is an attractive method for high-efficiency CTC isolation regardless of surface epitopes.

## Introduction

Hepatocellular carcinoma (HCC) is one of the most common malignancies in the world (Omata et al., 2017). In 1996, Mazzaferro et al. (1996) reported that liver transplantation (LT) was considered an optimal radical therapy for selected patients with HCC. By removing HCC tumors with the greatest possible margin and replacing cirrhotic liver tissue with a tumor-free graft, LT has a better curative effect for liver cancer than hepatectomy (Lee et al., 2010).

However, a significant proportion of HCC patients who undergo LT still suffer from unfavorable outcomes due to the chance of tumor recurrence (Trojan, Zangos & Schnitzbauer, 2016). One reason for the poor prognosis of liver cancer is that cancer cells can easily enter and spread through the bloodstream. Before surgical resection, these circulating tumor cells (CTCs) may be present in the patient’s blood. Surgical operations also carry the risk of causing CTCs to enter the bloodstream (Alix-Panabières & Pantel, 2014). Thus, CTCs are currently a major focus of liver cancer research because of the key roles they play in HCC recurrence and metastasis (Masuda et al., 2016; Kelley et al., 2015; Choi et al., 2015).

Several strategies have been used to separate blood for the analysis of CTCs. The most common enrichment approach, called immunoaffinity enrichment technology, such as applied by the CellSearch system, uses antibodies targeting epithelial cell adhesion molecule (EpCAM). This system is the only CTC isolation method currently approved by the US Food and Drug Administration for prognostic use. More recently, a number of approaches have been proposed for the isolation of CTCs using immunoaffinity selection, such as the CTC-chip (Nagrath et al., 2007), GEDI (Galletti et al., 2014), GEM (Sheng et al., 2014), and Abnova CytoQuest™ CR (Zhao et al., 2013; Hou et al., 2013; Wang et al., 2011), which still use EpCAM and other surface antigens as target moieties. However, CTCs may also undergo changes during disease states, such as epithelial–mesenchymal transition (EMT), accompanied by changes in CTC surface markers, leading to mesenchymal tumor cells with an increased stem-like phenotype and reduced epithelial phenotype (Giannelli et al., 2016). This may be why CTC detection rates and counts achieved using this enrichment technology are generally low and the entire population of CTCs cannot be collected (De Boer et al., 1999).

**Figure 1:**
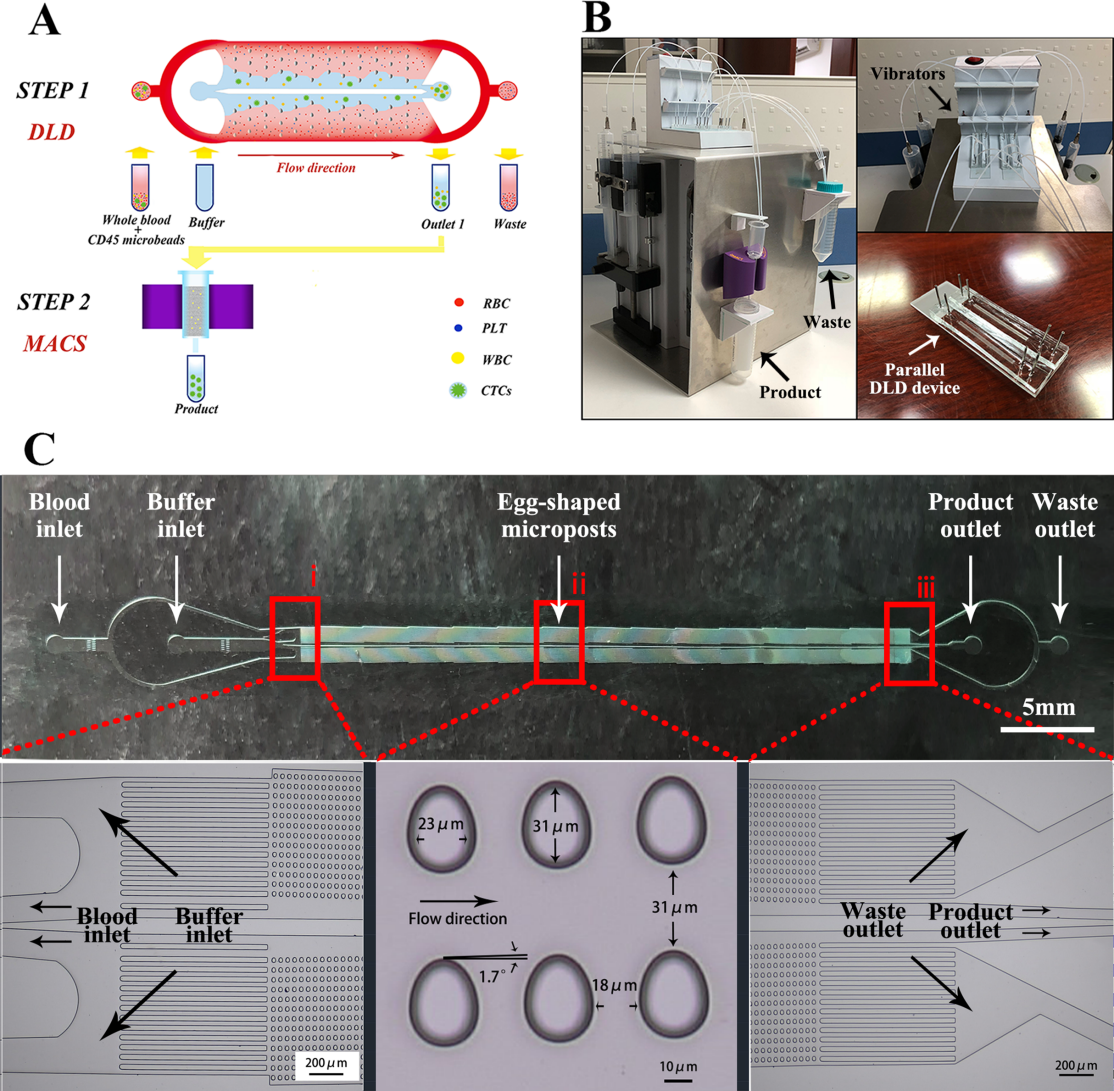
Platform schematic and structural design. (A) This capture platform integrates a DLD structure with a MACS separator for inline operation. Step 1: The DLD removes nucleated cells from whole blood by size-based deflection using a specially designed array of microposts. Step 2: The MACS separator serves to sensitively separate bead-labeled WBCs and unlabeled CTCs. (B) The overall system integrates syringe pumps, the DLD chip and the MACS separator into an operating platform. Before the sample enters the chip, a micro-oscillator mixes the blood to avoid stratification of the blood in the tube. (C) DLD device design. (i) Two inlets inject blood and buffer separately, the filter array ensures that blood flows along a certain direction; (ii) The device consists of two mirrored arrays of egg-shaped microposts with a pillar size and array offset designed to deflect particles larger than a certain size, thereby separating them from the main suspension; (iii) Two outlets are designed for the deflected product and undeflected waste. Photo credit: Xuebin Wang.

In this research, we attempted to combine emerging microfluidic technologies with magnetic-based cell sorting to generate a miniaturized, low-cost and antigen-independent tool for efficient CTC separation (Figs. 1A and 1B). Microfluidics, which integrates physical, chemical, and biological technologies at the microscale level, has been used to create devices known as labs-on-a-chip or micro total analysis systems. A new type of microfluidic structure using deterministic lateral displacement (DLD) was designed for the continuous high-throughput separating of whole blood from HCC patients to isolate nucleated cells, including CTCs and white blood cells (WBCs), efficiently with minimal damage and independent of antigens. Then, StraightFrom® Whole Blood CD45 MicroBeads and a magnetic-activated cell sorting (MACS) separator are used to remove bead-labeled WBCs from the DLD structure and ultimately concentrate unlabeled CTCs.

We also compared our platform with the Abnova CytoQuest™ CR system (Taipei, Taiwan) in separating and characterizing CTCs from HCC patients undergoing LT and performed follow-up analyses of HCC patients with early postoperative metastases. As CTCs from these samples underwent EMT, the detection sensitivity of the Abnova CytoQuest™ CR system was reduced, whereas our capture platform was not affected and yielded high CTC counts.

The results demonstrate that our capture platform is highly efficient independent from antigens and is thus clinically valuable.

## Materials and methods

### Deterministic lateral displacement microfluidic device design and function

The overall microfluidic DLD isolation platform is shown in Fig. 1C. This device is a size-based continuous-flow system because CTCs are generally larger in size than other blood cells (Harouaka, Nisic & Zheng, 2013). The system consists of an array of microposts with a pillar size and array offset designed to deflect particles above a certain size, thereby separating them from the main suspension (Karabacak et al., 2014). The design is based on the DLD theory proposed by Huang et al. (2004).

The flow chamber is 33 mm long, two mm wide, and 30 µm high. The microarray consists of two mirrored arrays of egg-shaped microposts, as shown in Fig. 1C, with the arrays at an angle of 1.7 ° with respect to the fluid flow direction. Based on the theoretical principle of DLD (Inglis et al., 2006), the critical particle diameter is approximately four to five µm. With this design, most of the red blood cells (RBCs) and platelets (PLTs) pass through the gaps and are removed through the outlet, while CTCs and some leukocytes are collected once they are concentrated.

### Deterministic lateral displacement microfluidic device fabrication

Microfluidic devices were fabricated using standard photolithography and soft lithography (Faustino et al., 2016). The negative photoresist SU-8 3025 (MicroChem, Naton, MA, USA) was used to fabricate the master on a silicon wafer with a photomask. Next, polydimethylsiloxane (PDMS) prepolymer mixed with a curing agent (8:1 w/w ratio) was poured onto the silicon wafer and baked at 80 °C for 1 h; subsequently, holes were punched for the inlet and outlets. Finally, the PDMS microfluidic channels and glass substrate were exposed to oxygen plasma (PDC-M; Chengdu Mingheng Science & Technology Co., Ltd., P. R. China) for 1 min and then brought into contact to bond together. The devices were left overnight in a 65 °C oven to complete the irreversible bonding process between the PDMS and glass.

### Magnetic-activated cell sorting separator and preparation

We used StraightFrom Whole Blood CD45 MicroBeads and a MACS separator (Miltenyi Biotec, Bergisch Gladbach, Germany) developed for the rapid selection of CD45^+^ cells directly from whole peripheral blood to remove bead-labeled WBCs and concentrate unlabeled CTCs. Unlike standard magnetic beads, Whole Blood CD45 MicroBeads have the advantage of not requiring sample preparation, such as density gradient centrifugation or erythrocyte lysis. MicroBeads (50 µl) were added to every one ml of anticoagulated whole blood. The samples were mixed well and incubated for 15 min in a refrigerator (2–8 °C).

### Platform module integration

The overall system is shown in Fig. 1B. The platform is driven by four syringe pumps. Two of the pumps are loaded with a 50-ml syringe containing 40 ml of prepared blood suspensions, and the remaining two pumps are loaded with a 50-ml syringe containing 50 ml of phosphate-buffered saline (PBS). Teflon tubes (one mm) connect the syringe pumps to the microfluidic device, and two small vibrators are located in front of the inlets. Some leukocytes and most cancer cells are deflected to the product outlet once they are concentrated, while the remaining blood, which is diluted with buffer, is collected via waste tubing. Collected cells drop down from the product outlet of the DLD device, which contains highly bead-labeled WBCs and CTCs, and enter the MACS column. Then, magnetically labeled CD45^+^ WBCs are retained within the column, while unlabeled cells (mostly CTCs) pass through the column and are collected at the final product outlet.

### Hepatocellular carcinoma cells

**Figure 2:**
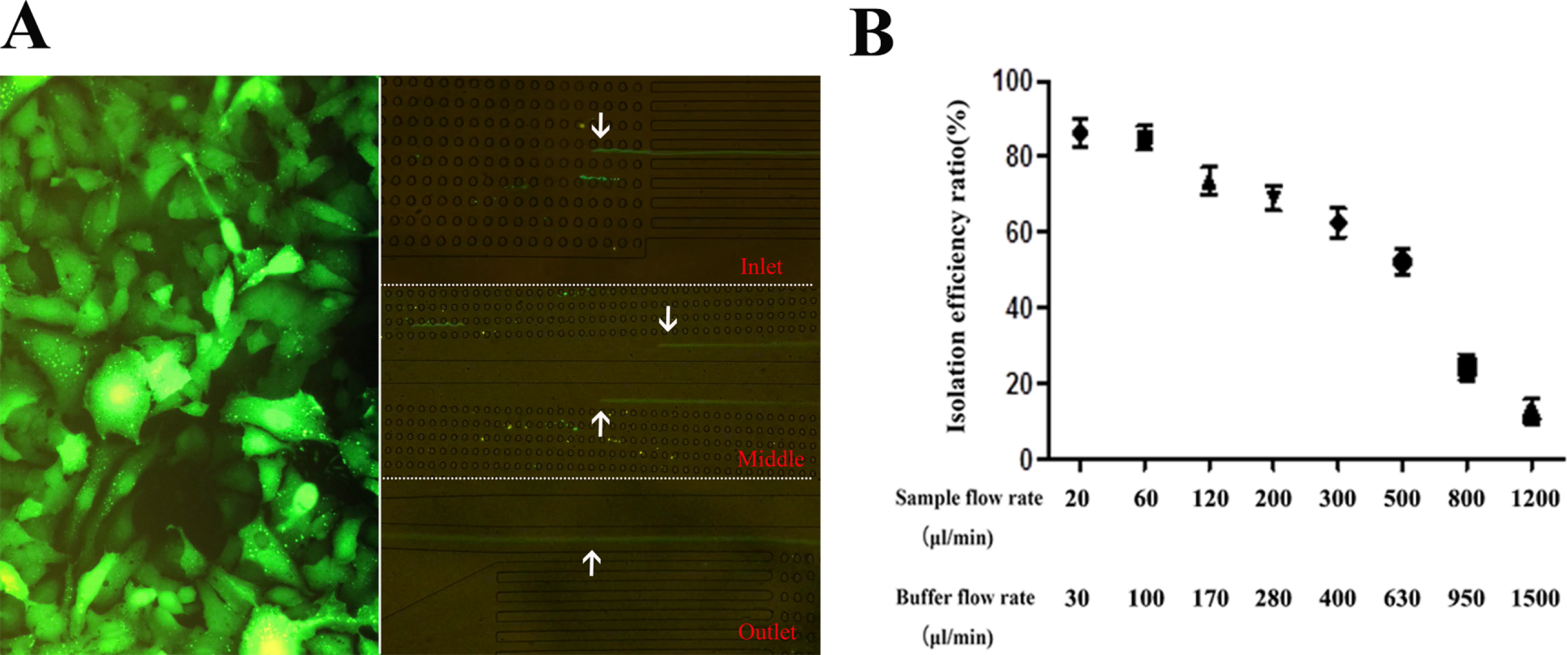
HepG2-GFP cells and isolation efficiency ratio with different flow rates. (A) Modeling the presence of cancer cells in human peripheral blood using HepG2-GFP cells. (B) Isolation efficiency ratio for different flow rates. A specimen flow rate of 60 μl/min and a buffer flow rate of 100 μl/min yielded an isolation efficiency ratio of 85.1 ± 3.2%.

To characterize the efficacy of the DLD device, we established HepG2 human liver cancer cells overexpressing green fluorescent protein (GFP) by lentiviral transfection (Fig. 2A), allowing the observation of these cells using a fluorescence microscope. Cells were cultured in low-glucose Dulbecco’s modified Eagle’s medium supplemented with 10% fetal bovine serum and 1% penicillin-streptomycin at 37 °C with 5% (v/v) CO_2_. The culture medium was changed every 2 days. Cells were harvested by incubation in 0.05% trypsin with 0.53 mM ethylenediaminetetraacetic acid (EDTA) at 37 °C for 3 min and were then diluted to the desired concentration.

### Blood samples and follow-up

Fresh whole blood samples were collected from 10 healthy volunteers and 12 HCC patients undergoing LT (Table 1) in the Transplant Section of the Beijing Friendship Hospital in China between September 2017 and May 2018. Blood was obtained prior to transplantation for HCC patients (baseline) and then at follow-up visits 1 and 3 months after the surgery. Recurrence was diagnosed based on typical imaging findings on computed tomography (CT), magnetic resonance imaging or bone scans. At each time point, 7.5 ml of fresh whole blood was collected into EDTA-containing tubes from each subject for analysis by our DLD platform and the CytoQuest™ CR system within 6 h. Clinical information was also collected, including the alpha-fetoprotein level, size, and number of tumors, Milan criteria (Mazzaferro et al., 1996), presence of hepatitis virus and vascular invasion.

**Table 1:** Patient characteristics.

Variables	Liver transplantation (*n* = 12) (%)
Sex	
Male	8(66.7)
Female	4(33.3)
Age, years	
≤50	7(58.3)
>50	5(41.7)
Etiology	
Hepatitis B	9(75)
Other	3(25)
Tumor number	
Single	5(41.7)
Multiple	7(58.3)
Tumor size, cm	
≤5	3(25)
>50	9(75)
Vascular invasion	
No	6(50)
Yes	6(50)
Milan criteria	
Within	4(33.3)
Beyond	8(66.7)

Written informed consent was obtained from all patients. Approval from the Beijing Friendship Hospital Human Research Ethics Committee (code: 2018-P2-114-01) was obtained for the use of all samples using a protocol that conforms to the provisions of the Declaration of Helsinki.

### Isolation efficiency of the platform integrating DLD with MACS separation

To determine the best flow rates and obtain the best isolation efficiency ratio from the device, we spiked a small number of HepG2-GFP cells into blood samples to model the presence of cancer cells in human peripheral blood. Blood samples provided by healthy volunteers were collected into EDTA-containing tubes and diluted 10 times with PBS to prevent channel blocking. HepG2-GFP cells were then spiked into the diluted blood specimens at 1 × 10^2^ cells/ml.

The sample and buffer were injected into the specimen and buffer inlets, respectively, at different flow rates, following the principle that the sample liquid level and the buffer liquid level would meet at the center of the chip. We counted the HepG2-GFP cells using a fluorescence microscope to select the best flow rates, and we then obtained the isolation efficiency ratio, *R*, using the equation $R = {C_1}{V_1}/\left( {{C_1}{V_1} + {C_2}{V_2}} \right)$, where *C*_1_ and *V*_1_ denote the concentration and solution volume of the final product outlet, respectively, while *C*_2_ and *V*_2_ denote the respective concentration and solution volume of the waste outlet. The experiment was repeated 10 times.

### HepG2-GFP cell sorting experiments

To examine whether there were differences in the enrichment effects of different concentrations of HCC cells, we spiked HepG2-GFP cells into 10-fold diluted whole blood at 50, 1 × 10^2^, 2 × 10^2^, 4 × 10^2^, and 8 × 10^2^ cells/ml. At the best flow rates, we recalculated the isolation efficiency as described above. Each concentration was tested 10 times.

### Debulking efficiency

To characterize the performance of the platform in removing blood cells (mainly RBCs, PLTs, WBCs), we spiked HepG2-GFP cells into 10-fold diluted whole blood at 1 × 10^2^ cells/ml and injected them into the device under the best flow rates. After the device was running stably, we calculated the removal ratio *R_r_* using the equation ${R_r} = \left( {{C_2}{V_2} - {C_1}{V_1}} \right)/{C_2}{V_2}$, where *C*_1_ and *V*_1_ denote the concentration and solution volume of the final product outlet, respectively, while *C*_2_ and *V*_2_ denote the respective concentration and solution volume of the starting sample. A hemocytometer was used to count blood cells, and the experiment was repeated 10 times.

### Abnova CytoQuest™ CR system

The CytoQuest™ CR system is a rare cell sorting system developed by Abnova and the UCLA (University of California Los Angeles) Nano Research Institute. The system is a modified NanoVelcro Chip that is capable of efficiently capturing CTCs in patient blood samples using anti-EpCAM-coated silicon nanowire substrates. Circulating tumor cell enumeration by CytoQuest™ CR was carried out according to the manufacturer’s protocol (Wang et al., 2011; Zhao et al., 2013; Hou et al., 2013). First, 7.5 ml of blood was drawn into an EDTA-containing collection tube. Next, the buffy coat containing CTCs was separated by gradient cell separation. After washing with PBS, the prepared buffy coat was injected into an EpCAM-coated microfluidic slide to capture CTCs.

### Immunostaining and identification of captured cells

Circulating tumor cells were identified and counted manually. After the produced sample was transferred to a 50-ml centrifuge tube and centrifuged at 600*×g* for 5 min, the supernatant was aspirated. Then, the cell pellet was diluted to five ml by PBS and divided equally on 10 glass slides (Thermo Fisher Scientific, Waltham, MA, USA).

Cells on the glass slides were fixed with 4% formaldehyde for 15 min at room temperature and then permeabilized with 0.1% Triton X-100 in PBS. To identify CTCs, double immunofluorescence staining was performed with anti-cytokeratin-fluorescein isothiocyanate (anti-CK-FITC; Miltenyi Biotec, Bergisch Gladbach, Germany) and anti-CD45- allophycocyanin (anti-CD45-APC; Miltenyi Biotec, Bergisch Gladbach, Germany). 4′,6-Diamidino-2-phenylindole (DAPI; Sigma Aldrich, St. Louis, MO, USA) was used to stain nuclei. To identify CTCs undergoing EMT, cells were stained with anti-vimentin- phycoerythrin (anti-vimentin-PE; Miltenyi Biotec, Bergisch Gladbach, Germany). Cells were counted using confocal laser scanning microscopy (Lecia, Wetzlar, Germany).

The criteria for WBCs were as follows: round/ovoid, DAPI^+^/CD45^+^/CK^−^ cells ≤6 µm in size. Circulating tumor cells were defined as round/ovoid DAPI^+^/CD45^−^/CK^+^ cells ≥6 µm in size. Circulating tumor cells undergoing EMT were defined as round/ovoid, DAPI^+^/CD45^−^/CK^+^/vimentin^+^ cells ≥6 µm in size (Court et al., 2018; Yu et al., 2016).

### Statistical analysis

All statistical analyses were performed using SPSS (version 22.0; IBM Corp., Armonk, NY, USA). Variance analysis was used to examine whether the isolation efficiency varied with cell concentration. A pairwise comparison between CTCs counted at different time points after detection by our integrated DLD platform and the CytoQuest™ CR system was performed by applying the nonparametric Wilcoxon signed-rank test. Graphical plots were generated using GraphPad Prism 6.0 (GraphPad Software, La Jolla, CA, USA). All *P*-values were two-sided, and *P-*values of less than 0.05 were considered statistically significant.

## Results

### Isolation efficiency and optimal flow rates for the platform integrating DLD with MACS separation

HepG2-GFP cells were spiked into 10-fold diluted whole blood at a concentration of 1 × 10^2^ cells/ml. At different sample flow rates of 20, 60, 120, 200, 300, 500, 800, and 1,200 μl/min, buffer was injected into the device at flow rates of 30, 100, 170, 280, 400, 630, 950, and 1,500 μl/min. The corresponding isolation efficiency ratios were 86.4% ± 3.7% (mean ± SD), 85.1% ± 3.2%, 73.7% ± 3.5%, 69.0% ± 3.3%, 62.5% ± 3.8%, 52.2% ± 3.5%, 24.3% ± 3.9%, and 12.6% ± 3.2%, respectively.

The cell isolation efficiency decreased significantly with increasing flow rate. To maintain a balance between isolation efficiency and time, we selected a specimen flow rate of 60 μl/min and a buffer flow rate of 100 μl/min, which yielded an isolation efficiency ratio of approximately 85.1% ± 3.2%, as shown in Fig. 2B.

**Figure 3:**
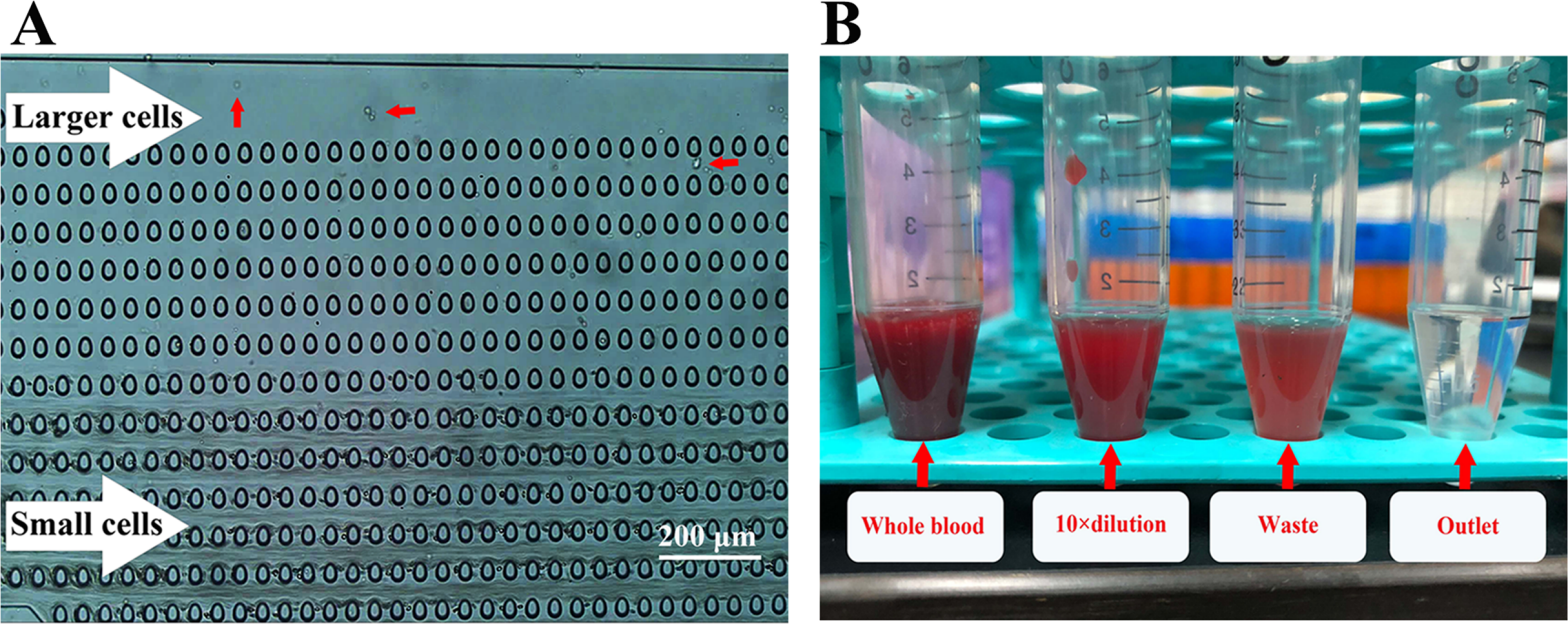
DLD platform enrichment experiment using blood samples. (A) The flow of blood in the chip observed through an optical microscope. Small cells, mainly composed of RBCs, flowed in a straight line, while large cells flowed at an angle and separated from the main suspension. (B) Samples obtained at different stages. Almost all RBCs were removed by the DLD chip. Photo credit: Xuebin Wang.

The flow of blood in the chip observed continuously through an optical microscope, as shown in Fig. 3A.

### Isolation efficiency with different concentrations of cultured HCC cells

We spiked HepG2-GFP cells into 10-fold diluted whole blood at 50, 1 × 10^2^, 2 × 10^2^, 4 × 10^2^, and 8 × 10^2^ cells/ml. At a specimen flow rate of 60 μl/min and a buffer flow rate of 100 μl/min, the isolation efficiency ratio was 85.2% ± 3.6%, 85.6% ± 3.3%, 84.8% ± 3.3%, 85.0% ± 3.1%, and 85.1% ± 3.5%, respectively. Analysis of variance suggested no significant differences between the different groups (*P* = 0.972 > 0.05).

### Debulking efficiency and enrichment performance

The calculated results show that the RBC, WBC, and PLT removal rates were 99.90% ± 0.03%, 98.50% ± 0.13%, and 99.95% ± 0.05%, respectively. Significant changes in the appearance of the samples as shown in Fig. 3B. HepG2-GFP cells had 852 × enrichment over RBCs, 57 × enrichment over WBCs and 1,715 × enrichment over PLTs as illustrated in Table 2.

**Table 2:** Relative concentrations of erythrocytes, leukocytes, platelets, and HepG2-GFP recovered from the collected solution at a specimen flow rate of 60 μl/min and a buffer flow rate of 100 μl/min.

	Concentration (%)			
	Erythrocytes	Leukocytes	Platelets	HepG2-GFP
Sample	100	100	100	100
Collected solution	0.100 ± 0.015	1.500 ± 0.017	0.050 ± 0.003	85.100 ± 3.213

### Patient characteristics and follow-up

**Figure 4:**
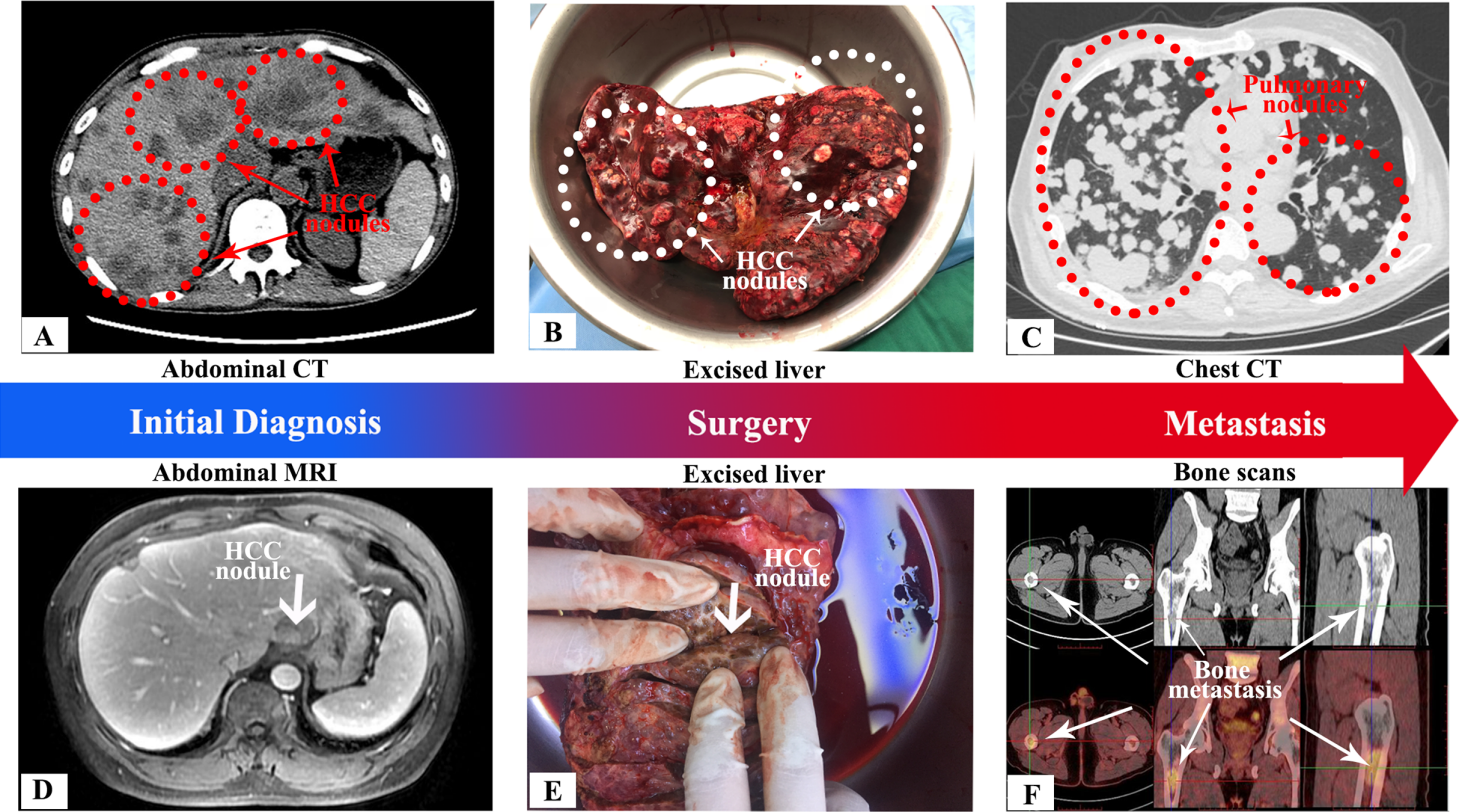
Preoperative CT and MRI scans, intraoperative tumor images and evidence of postoperative metastasis for two patients with tumor metastases. (A), (B), and (C) showed HCC patient no. 1. (A) Axial portal venous phase CT showed multiple, ill-defined lesions in liver (red line). The multiple lesions were presented hypodense, relative to the surrounding liver, in portal venous phase; (B) HCC nodules removed during surgery (white line); (C) Axial chest CT showed multiple, ill-defined, oval nodules in lungs (red line). The nodules were distributed at random. Combined with the clinical history, the lesions were considered as metastasis. (D), (E), and (F) showed HCC patient no. 2. (D) Axial arterial phase TIWI C^+^ showed well-defined, focal lesion in liver (arrow). The lesion was presented heterogeneous hypointense enhancement in later arterial phase; (E) HCC nodules removed during surgery (arrow); (F) Bone scans showed a focal lesion with hypermetabolism in right femoral cavity (arrow). There was no obvious bone destruction around the lesion. Based on the clinical history, the lesion was considered as metastasis. Photo credit: Xuebin Wang.

Among the 12 enrolled patients with HCC, 7/12 (58.3%) had multiple tumors, 9/12 (75%) had a tumor size greater than five cm, 8/12 (66.7%) extended the Milan criteria, and 6/12 (50%) had vascular invasion. Hepatocellular carcinoma recurrence after LT was observed in two patients (16.7%) during follow-up. One example of metastasis is illustrated in Fig. 4A. The patient was a 65-year-old male with diagnoses of hepatitis B cirrhosis and HCC. He underwent LT, which revealed a 20-cm dominant lesion with multiple satellite lesions and microvascular invasion. However, he was found to have multifocal lung nodules on his 3-month follow-up CT scan and then died from respiratory failure. Another example of metastasis is illustrated in Fig. 4B. The patient was a 49-year-old male with a history of hepatitis B cirrhosis and HCC who had undergone radical hepatectomy 1 year prior. Due to the recurrence of HCC, he underwent LT, which revealed a three-cm dominant lesion. At 2 months after LT, bone scans indicated bone metastasis.

Ten healthy volunteers were followed for an equivalent amount of time. None of the healthy control patients involved in the study developed HCC during follow-up.

### Follow-up detection of CTCs using our DLD platform and the CytoQuest™ CR system

We tested the clinical feasibility of using our device to isolate CTCs from preoperative HCC patients and healthy volunteers. We successfully collected CTCs from all HCC patients (100%), while a single CTC was found in 2/10 (20%) healthy control volunteers, representing a false positive.

The number of CTCs detected in patients by our integrated platform and the CytoQuest™ CR system is shown in Table 3. Among patients with no metastasis, no significant differences in the CTC detection results were found at the three-time points by the Wilcoxon signed-rank test (preoperation: *P* = 0.472 > 0.05; 1 month postoperation: *P* = 0.145 > 0.05; 3 months postoperation: *P* = 0.395 > 0.05). However, among patients with metastasis, significantly different CTC counts were detected by our DLD platform and the CytoQuest™ CR system.

**Table 3:** The number of CTCs in every 7.5 ml of whole blood from patients detected by the DLD integrated platform and CytoQuest™ CR system.

No. of patients	No metastasis										Metastasis	
	1	2	3	4	5	6	7	8	9	10	1	2
Preoperation												
DLD platform CTC counts	30	11	9	12	28	14	22	26	10	36	35	25
CytoQuest™ CR CTC counts	36	9	4	13	25	16	20	25	7	38	1	5
1 month postoperation												
DLD platform CTC counts	7	4	5	9	7	3	11	10	4	10	15	10
CytoQuest™ CR CTC counts	5	1	2	6	5	5	12	13	2	8	5	6
3 months postoperation												
DLD platform CTC counts	40	5	3	11	10	7	12	9	6	5	30	20
CytoQuest™ CR CTC counts	34	5	2	9	11	4	20	9	6	4	19	15

Examination of the two metastatic patients using the CytoQuest™ CR system before and after LT revealed relatively low numbers of CTCs (preoperation: 1 and 5; 1 month postoperation: 5 and 6; 3 months postoperation: 19 and 15), whereas our DLD integrated platform collected significantly more cells (preoperation: 35 and 25; 1 month postoperation: 15 and 10; 3 months postoperation: 30 and 20).

### Detection of vimentin^+^ CTCs

**Figure 5:**
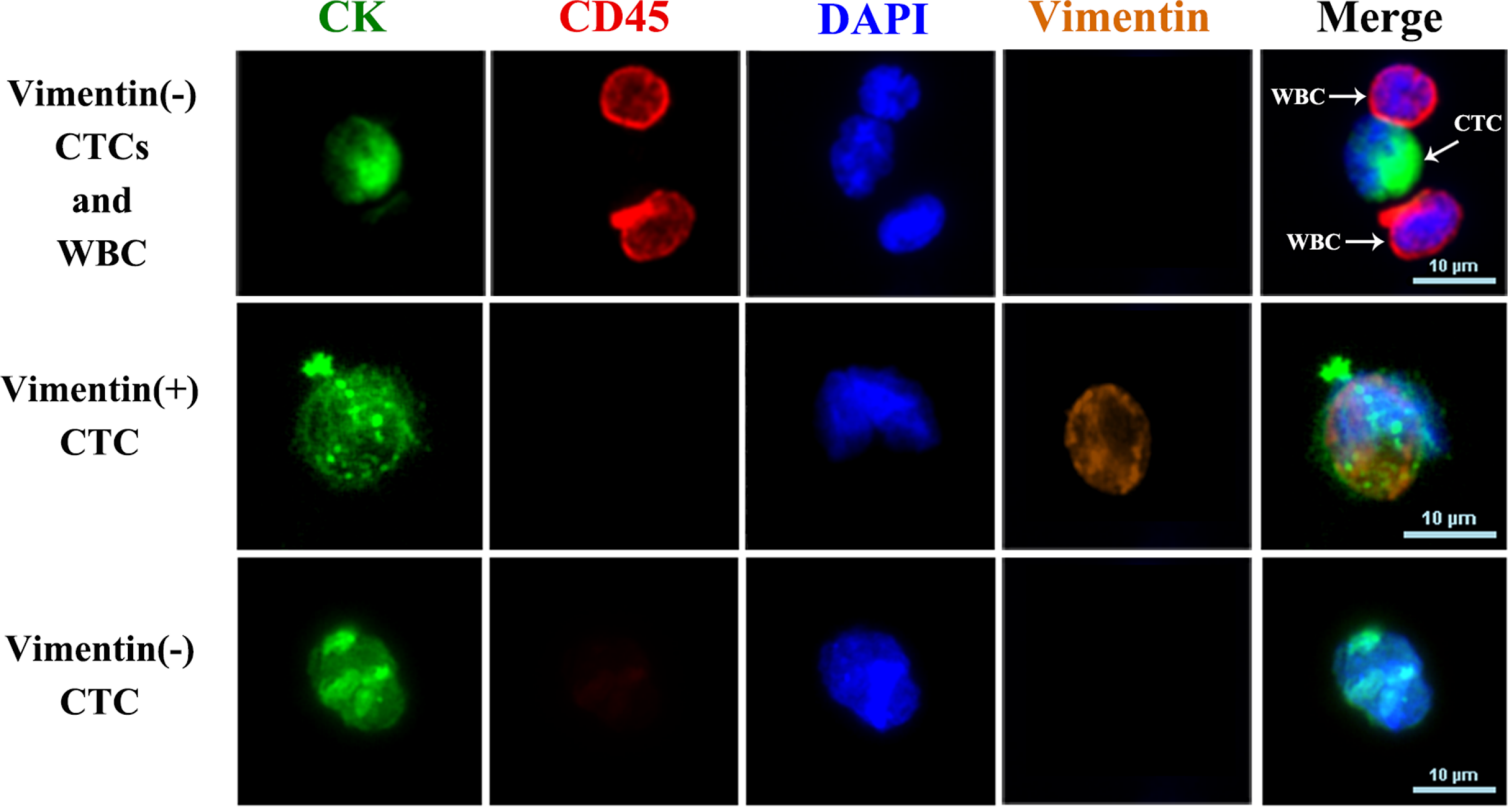
Fluorescence images of CTCs and WBCs. Immunofluorescence images of cells stained with antibodies against CK (green, CTC marker), CD45 (red, leukocyte marker), and DAPI (nuclear stain, blue). Anti-vimentin antibody (orange) was used to identify CTCs undergoing EMT.

**Figure 6:**
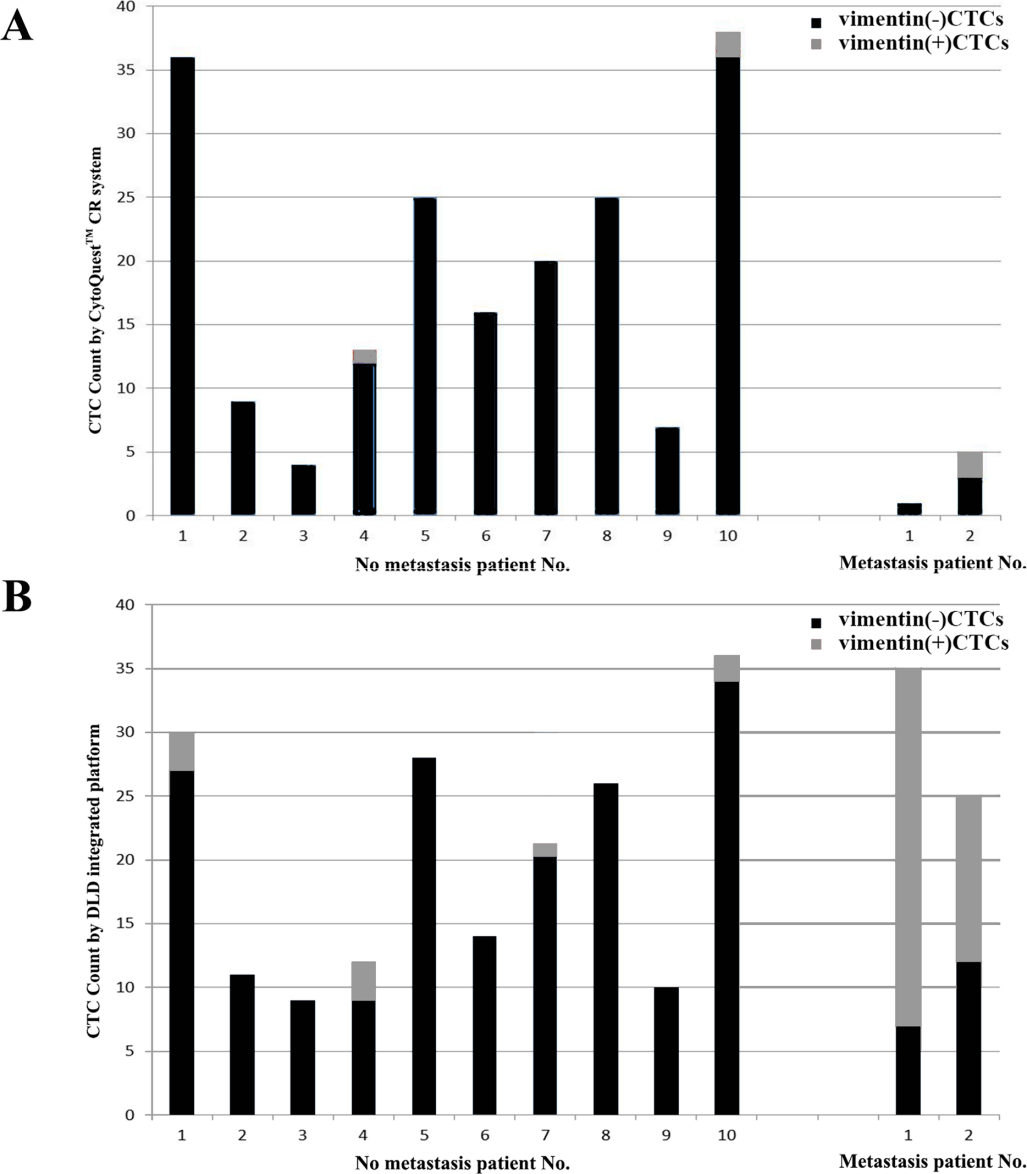
Numbers of CTCs that were enriched by the DLD integrated platform and CytoQuest™ CR system in HCC patients before surgery, including different subpopulations of vimentin^+^ and vimentin-cells. (A) Numbers of CTCs that were enriched by CytoQuest™ CR system. (B) Numbers of CTCs that were enriched by the DLD integrated platform.

We further analyzed the CTCs extracted by our DLD integrated platform and the CytoQuest™ CR system before surgery by examining the expression of vimentin (Fig. 5), an EMT marker (Satelli & Li, 2011). As shown in Table 4 and Fig. 6, immunostaining showed that vimentin^+^ CTCs were found in 4/10 (40%) and 2/10 (20%) patients without metastasis by our DLD integrated platform and the CytoQuest™ CR system, respectively, and the counts were low.

**Table 4:** The number of vimentin^−^ and vimentin^+^ CTCs in every 7.5 ml of whole blood from patients before surgery detected by the DLD integrated platform and CytoQuest™ CR system.

No. of patients	No metastasis										Metastasis	
	1	2	3	4	5	6	7	8	9	10	1	2
DLD platform												
Vimentin^−^ CTCs	27	11	9	9	28	14	20	25	10	36	7	12
Vimentin^+^ CTCs	3	0	0	3	0	0	1	1	0	0	28	13
Total	30	11	9	12	28	14	21	26	10	36	35	25
CytoQuest™ CR system												
Vimentin^−^ CTCs	36	9	4	12	25	16	20	25	7	36	1	3
Vimentin^+^ CTCs	0	0	0	1	0	0	0	0	0	2	0	2
Total	36	9	4	13	25	16	20	25	7	38	1	5

## Discussion

Liver transplantation is used for the complete removal of tumor lesions, and the tumor recurrence and metastasis observed in HCC patients is likely related to CTCs. Therefore, the determination of CTCs by “liquid biopsies” is currently a major focus of investigation in monitoring patients with HCC undergoing LT.

In the current study, we describe a platform that integrates microfluidic DLD arrays with a MACS separator and demonstrates its great potential for tumor antigen-independent CTC separation. At the optimal specimen flow rate of 60 μl/min and buffer flow rate of 100 μl/min, a CTC isolation efficiency ratio of 85.1 ± 3.2% and a high blood cell debulking efficiency (RBC, PLT, and WBC removal rates of 99.90% ± 0.03%, 99.95% ± 0.05%, and 98.50% ± 0.13%, respectively) were achieved. In addition, there were no significant difference in the isolation performance of different tumor cell concentrations ranging from 50 to 8 × 10^2^ cells/ml.

Furthermore, the DLD device can provide continuous CTC separation. StraightFrom Whole Blood CD45 MicroBeads have the advantage of not requiring sample preparation for the rapid selection of CD45^+^ cells directly from whole peripheral blood; thus, the cells produced from the DLD device can enter the MACS separator directly without centrifugation. Therefore, compared with other currently widely used methods for the negative selection of CTCs, such as RBC lysis or density gradient centrifugation, our capture platform has the advantage of simple operation.

At the 3-month follow-up of the 12 HCC patients undergoing LT, CTCs were found in 12 (100%) patients. Ten of the 12 patients (83.3%) did not exhibit tumor metastasis or recurrence. Two of the 12 patients (16.7%) were diagnosed with metastasis, with recurrence in the lungs (*n* = 1) and bone (*n* = 1).

Compared with the Abnova CytoQuest™ CR system, our platform yielded similar results among patients with no metastasis, as determined by the Wilcoxon signed-rank test. However, examination of the two metastatic patients using the CytoQuest™ CR system before and LT revealed relatively fewer CTCs than examination by our platform.

Our results suggest the identification of patients with tumor metastases and subpopulations of EMT-transformed CTCs. Previous studies have shown that CTCs undergo dynamic evolution under the strong selective pressure of chemotherapy, radiation therapy and any other therapeutic intervention (Aktipis et al., 2011). These CTCs have lower EpCAM expression (Giannelli et al., 2016) and increased metastatic potential (Mani et al., 2008). Therefore, they are difficult to detect, isolate, and characterize using immunoaffinity enrichment strategies (De Boer et al., 1999), and EpCAM^low^ CTCs can be missed by the CytoQuest™ CR bulk sorting approach. In contrast, our antigen-independent platform maintained a high detection sensitivity while achieving higher CTC counts consistent with clinical observations. However, given that the sample size is relatively small, larger studies to confirm the present results are needed.

We also observed limitations of the experiments. The DLD device uses a size-based particle separation approach, but tumor cells have different diameters. Although most CTCs are larger than RBCs, there are still a small number of CTCs that are similar in diameter to RBCs (Fachin et al., 2017). These cells would not be separated by the device and would eventually be lost. In addition, although CTCs were highly enriched by our platform (HepG2-GFP cells had 852 × enrichment over RBCs, 57 × enrichment over WBCs, and 1,715 × enrichment over PLTs), the remaining cells still affect the CTC extraction purity. Finally, there is a delicate relationship between the flow rate and the separation efficiency. If researchers want to separate large volumes of blood samples at a high rate, cells under a high shear stress (Liu et al., 2013) can deform because of the high fluid velocities in our device, which may influence the cell isolation efficiency.

In future studies, we will continue to improve the CTC extraction purity. In addition, using our platform, we will collect CTCs for further cell culture, drug screening, and molecular analyses.

## Conclusions

Here, we demonstrate the successful use of a platform that integrates a DLD structure with a MACS separator for isolating CTC isolation with high-efficiency regardless of surface epitopes. Compared with the CytoQuest™ CR system, which is based on an immunoaffinity method, our platform successfully identified a distinct subpopulation of EMT-transformed, vimentin^+^ CTCs.

However, the device still has several limitations, as described above. Our future studies will focus on continuing to improve the device.

## Additional Information and Declarations

### Data Availability

The following information was supplied regarding data availability: The raw data are available in the figures and tables in the article.
